# The Prevalence and Negative Impacts of Substance Use Disorders among People with HIV in the United States: A Real-Time Delphi Survey of Key Stakeholders

**DOI:** 10.1007/s10461-021-03473-9

**Published:** 2021-09-29

**Authors:** Bryan R. Garner, Heather J. Gotham, Hannah K. Knudsen, Brittany A. Zulkiewicz, Stephen J. Tueller, Marcus Berzofsky, Tom Donohoe, Erika G. Martin, L. Lauren Brown, Theodore Gordon

**Affiliations:** 1 RTI International, Research Triangle Park, P. O. Box 12194, Durham, NC 27709 USA; 2 Stanford University School of Medicine, 1520 Page Mill Road MC 5265, Palo Alto, CA 94304 USA; 3 University of Kentucky, 845 Angliana Avenue, Room 204, Lexington, KY 40508 USA; 4 Pacific AIDS Education and Training Center, University of California Los Angeles, Los Angeles, CA 90024 USA; 5 Rockefeller College of Public Affairs and Policy at the University at Albany, Both part of the State University of New York, 1400 Washington Avenue, Milne 300E, Albany, NY 12222 USA; 6 Department of Psychiatry and Behavioral Sciences, School of Medicine, Meharry Medical College, Nashville, TN USA; 7 Infectious Disease Division, Department of Medicine, Vanderbilt University Medical Center, Nashville, TN USA; 8 TJG, LLC, 1 Smilax Dr, Old Lyme, CT 06371 USA

**Keywords:** HIV, Substance use disorders, Addiction treatment, Integrated care

## Abstract

Although HIV and substance use disorders (SUDs) constitute a health syndemic, no research to date has examined the perceived negative impacts of different SUDs for people with HIV (PWH). In May 2019, 643 stakeholders in the U.S., representing clients of AIDS service organizations (ASOs), ASO staff, and HIV/AIDS Planning Council members, participated in an innovative Stakeholder-Engaged Real-Time Delphi (SE-RTD) survey focused on the prevalence and individual-level negative impact of five SUDs for PWH. The SE-RTD method has advantages over conventional survey methods by efficiently sharing information, thereby reducing the likelihood that between-group differences are simply due to lack of information, knowledge, and/or understanding. The population-level negative impacts were calculated by weighting each SUD’s individual-level negative impact on indicators of the HIV Care Continuum and other important areas of life by the perceived prevalence of each SUD. Overall, we found these SUDs to have the greatest population-level negative impact scores (possible range 0–24): alcohol use disorder (population-level negative impact = 6.9; perceived prevalence = 41.9%), methamphetamine use disorder (population-level negative impact = 6.5; perceived prevalence = 3.2%), and opioid use disorder (population-level negative impact = 6.4; perceived prevalence = 34.6%). Beyond further demonstration of the need to better integrate SUD services within HIV settings, our findings may help inform how finite funding is allocated for addressing the HIV-SUD syndemic within the U.S. Based on our findings, such future efforts should prioritize the integration of evidence-based treatments that help address use disorders for alcohol, methamphetamine, and opioids.

## Introduction

The HIV pandemic remains a major public health challenge within the United States (U.S.) and globally [1]. In 2019, the U.S. launched a new initiative called *Ending the HIV Epidemic: A Plan for America*, which aims to end HIV in the U.S. by 2030 [2]. However, substance use disorders (SUD) among people with HIV (PWH) are prevalent [3–5] and can negatively impact PWH progressing successfully along the HIV care continuum [6–13]. In fact, HIV and substance use disorders (SUD) constitute a health syndemic [5], and it was recently noted by the Director of the National Institute on Drug Abuse (NIDA) that, “the ambitious goal of ending the transmission of HIV will never be realized if we do not also address drug use while ensuring there are no disparities in access to treatment of HIV and SUD [14].”

One of the keys to addressing SUD among PWH is to integrate SUD services within community-based AIDS Service Organizations (hereafter referred to as ASOs) across the U.S., including screening, referral to specialty SUD treatment, onsite pharmacological and psychosocial treatments, and SUD-related wraparound services [15]. Integrated services, where ASOs deliver SUD services on-site, result in better patient outcomes and are cost-effective [16, 17]. Nonetheless, despite more than a decade of calls to improve SUD service integration within ASOs, the need for improvement remains urgent [18–21]. Having the best understanding possible regarding the current intersection of HIV and SUDs may assist ASOs and their staff in prioritizing their finite resources moving forward. This includes understanding there are multiple evidence-based treatment interventions available, yet some are only appropriate for certain SUDs [22]. Furthermore, there are policy implications. Similar to how increased recognition of the U.S. opioid epidemic contributed to an enormous influx of new funding for addressing opioid use disorders [23], increasing recognition of the second wave of the methamphetamine epidemic [24] may contribute to new policies and funding to help address methamphetamine use disorders among people with and without HIV.

Previous research has documented elevated rates of SUD among PWH. Using data from PWH served by seven academic medical centers in the Northeast, South, and West between 2007 and 2014, Hartzler et al. [5] found that 48% had a SUD, which is 6.5 times greater than the general U.S. population. Specifically, Hartzler and colleagues found the highest-to-lowest prevalence for five substances to be 31% for marijuana use disorder, 19% for alcohol use disorder, 13% for methamphetamine use disorder, 11% for cocaine use disorder, and 4% for opioid use disorder. Alarming as these rates may be, population-based rates of SUD in PWH may be even greater. Indeed, based on a recent systematic review and meta-analysis, Duko et al. [25] reported an estimated 42% prevalence of alcohol use disorder among PWH in developed countries including the U.S. Differences in rates of SUD among PWH in these studies may be due to multiple factors including regional differences, changes in substance use patterns over time [26, 27], and other study limitations. Thus, there is currently a need to better understand how prevalent specific SUDs are among PWH from broader samples than surveys of PWH treated in academic medical centers.

Notwithstanding its public health importance, prevalence is only one element in considering the possible negative impact associated with SUDs. Another important component is the differential negative impact of each SUD. Although focused on substances rather than SUDs, across three studies using similar expert ratings of harm to others and harm to the person using the substance, alcohol (not alcohol use disorder) was consistently identified as the most harmful substance. Although the perceived harm to the person using alcohol was similar to that for other substances, the perceived harm to others through injury and accidental death, family problems, and economic and community impacts was quite high compared to other substances. Perceptions regarding the harmful impacts of other substances were less consistent [28–30]. For example, in the United Kingdom and European Union, heroin and crack cocaine were identified as the second and third most harmful substances behind alcohol [29, 30]. For heroin, harms related to crime and mortality were identified as important harms, while crime, physical dependence, and impairment in cognitive functioning were identified as important harms for crack cocaine [29]. However, in Australia, using prevalence rates as weights, the rankings of the five most harmful substances (in order) were alcohol, tobacco, methamphetamines, marijuana, and opioids [28]. For those substances other than alcohol, mortality and other health problems, as well as loss of livelihood and relationships, were large negative consequences.

Although some previous research has examined national perceptions of harm or risk from substance use [31], to the best of our knowledge no research to date has reported the perceived impact across different SUDs in the U.S. general population or for PWH (both in the U.S. or elsewhere). Thus, our objectives are threefold: (1) to examine the prevalence of five SUDs among PWH in the U.S., (2) to measure perceived negative impacts for PWH who have these SUDs, and then by weighting the individual-level impacts by prevalence, (3) to estimate the population-level negative impacts of these five SUDs among PWH. As noted above, such data have the potential for significant effects on policy and funding regarding how to best address comorbid HIV and SUD, which will assist in ending the HIV epidemic. Furthermore, the likelihood of such positive effects resulting from our research have been enhanced by incorporating methodological improvements offered by Dubljevic [32], such as including multiple stakeholder perspectives, focusing on a smaller subset of substances, and separating SUDs from substance use. More specifically, we focused on the perspectives of three key stakeholder groups (clients with HIV, ASO staff, and HIV planning council representatives), the five substances examined by Hartzler and colleagues, and SUDs (as opposed to substance use). The current research was considered exploratory, and no specific hypotheses were made. However, for each of the three objectives, there was an a priori interest in examining the extent to which there were differences by U.S. region (i.e. Northeast, South, Midwest, West) and by stakeholder perspective (i.e. client with HIV, ASO organization staff, HIV planning council representative).

## Methods

### Study Design

We used a cross-sectional observational design, with all participants being asked to report their current perceptions regarding SUD prevalence among PWH in their geographic area and individual-level negative impacts of that type of SUD on four HIV care continuum indicators and four other important areas of life (see below for more details). Although a traditional cross-sectional survey would have been much easier to conduct, we chose a Stakeholder Real-Time Delphi survey to enable each stakeholder participant to anonymously share their reason(s) for their response(s), review other participant’s responses and reasons, and have the opportunity to change their response and reasons (i.e. participants may wish to change their response and/or reasons based on learning new information shared by other participants).

Delphi surveys have been used to collect expert opinion since the 1960s and offer a ground up approach to study an issue or problem [33]. Developed at RAND as a systematic means for consensus seeking among selected groups, Delphi surveys have been used in studies across a wide range of topics and are frequently used in academic settings. The original Delphi survey design included a series of sequential questionnaires; reasons for extreme positions obtained in an early questionnaire were fed back to participants in subsequent questionnaires with a request for reconsideration of prior answers. Anonymity was offered to participants to reduce chances for reputation bias [33]. Despite the success of such studies, they were expensive and time consuming. A variation known as Real-Time Delphi was introduced by Gordon and Pease [34]. This method preserved the key principles of conventional Delphi surveys, which are: (1) anonymity, (2) controlled feedback of responses to all group members, (3) iteration, and (4) statistical aggregation of individuals' responses) [35]. Real-Time Delphi surveys have been shown to produce results similar to conventional Delphi surveys, but more quickly and efficiently due to feedback being provided immediately (i.e. in real time) [36].

### Setting and Participants

First, research staff developed a comprehensive database of non-clinical community-based ASOs and their key contacts via several existing resources (e.g. HIV.gov, poz.com, thebody.com), and then called each ASO to confirm they were still active and to update contact information. Then, between March and April 2019, all known and active ASOs and HIV planning councils (hereafter referred to as HPCs) within the U.S. were recruited by email invitation to consider participation in a study funded by NIDA that focused on better understanding the prevalence and negative impacts of five SUDs (alcohol, cannabis/marijuana, cocaine, methamphetamine, and opioids). We sought to recruit 3–5 clients per ASO, 3–5 staff per ASO, and 3–5 members per HPC. After providing online informed consent, each participant completed a brief online screener, which included questions about their background characteristics and experiences with SUDs. To be eligible for the interactive survey, participants had to report in the screener: (a) being at least 18 years of age, (b) having personal, professional (e.g. clients), and/or other experience with SUDs (e.g. have/had a family member, significant other, or close friend with a SUD) for at least one of the five substances of interest, (c) residing in the United States, and, (d) if they reported being an ASO client, being HIV-positive. To ensure participants had at least some relevant SUD experience, we planned to exclude individuals without any personal and/or other experience with SUDs (e.g. have/had a family member or significant other with a SUD, a close friend with a SUD, a client with a SUD) for any of the five substances of interest. However, none of the screened participants met this exclusion criteria. During a two-week period in May 2019, consenting and eligible participants were asked to complete an interactive survey. Each participant received a $50 gift card as compensation for their time to complete the interactive survey, defined as logging into the survey at least once in each of the two weeks.

### Data Source and Variables

Using SurveyGizmo’s secure online survey platform, an online participant screener form was used to collect background variables (i.e. age, gender identity, ethnicity, and race), variables regarding HIV status (i.e. ever diagnosed) and past-year engagement in HIV care among individuals who had been diagnosed with HIV. Drawing on the work of Harzler et al. and after discussion by a coalition of stakeholders advising the research team, five substances were selected: alcohol, cannabis/marijuana, cocaine, methamphetamines, and opioids. Participants were asked to indicate if they had ever used each substance (i.e. lifetime use) or ever met two or more of the 11 Diagnostic and Statistical Manual of Mental Disorders (5th ed.; DSM–5) [37] SUD criteria during a 12-month period (i.e. lifetime SUD). Each participant was also asked to indicate if they had other experience related to the five SUDs (e.g. have/had a family member or significant other with the specific use disorder, have/had a close friend with the specific use disorder, have/had a client with the specific use disorder).

We customized a web-program developed as part of prior research [34, 38] for our project’s Real-Time Delphi survey. It comprised five main pages, one per substance. Each page presented the DSM-5 SUD criteria (e.g. spent a lot of time using the substance and/or recovering from use of the substance; taken the substance in large amounts or more often than they meant to; failed to meet responsibilities at work, school, or home because of their use of the substance; continued to use the substance even though they knew using the substance may have caused a physical or psychological problem to happen or get worse) and reminded respondents that presence of an SUD was indicated by meeting 2 or more of the criteria in the past 12 months. For each substance, participants were asked the following: “Thinking about people living with HIV in your area, please estimate the percentage that you believe have a use disorder for this substance (i.e. 2 or more of the 11 criteria during the past 12 months).” The perceived individual-level negative impact of each SUD was estimated by asking participants to think about PWH in their area and then rate the negative impact (if any) of having a SUD for each substance on the following: (1) being linked to HIV care, (2) being retained in HIV care, (3) being prescribed HIV medications, (4) being virally suppressed, (5) having stable housing, (6) having a reliable mode of transportation, (7) being employed, and (8) having a strong social support system. The first four items were drawn from the HIV Care Continuum [39], while the latter four items are key facilitators that increase the likelihood of achieving the elements of the care continuum [40–43]. Each item was rated using a 4-point scale (1 = no negative impact at all; 2 = a minor negative impact, 3 = a moderate negative impact; 4 = a major negative impact), which prior to analyses was recoded (i.e. 0 = no negative impact at all; 1 = a minor negative impact, 2 = a moderate negative impact; 3 = a major negative impact). For each SUD, an individual-level negative impact score was computed by taking the sum of the eight items (possible range from 0 to 24). Additionally, a population-level negative impact score (possible range also 0–24) was computed for each SUD by weighting each individual-level negative impact score by the proportion (e.g. 0.419 = 41.9%) of PWH perceived to have that SUD. After submitting their responses, participants were immediately able to see the mean responses from all other study participants. Participants logged into the survey at least one more time in the following week to revise their responses.

### Bias

The primary way we sought to minimize bias was through our recruitment approach, in which we developed a comprehensive database of all ASOs in the U.S. (*n* = 664) and then invited each to have their respective staff (both leadership and direct care staff) and clients consider study participation. Additionally, all HPCs (*N* = 52) in the U.S. were invited to have their members consider participation. However, because we purposively sought participants that indicated having at least some SUD-related knowledge and/or experience via the participant screener, our sample may not represent the perceptions of all clients with HIV and staff, or all HPC members.

### Statistical Methods

Our analysis had three key outcomes for each of the five substances: (1) use disorder prevalence, (2) individual-level negative impact, and (3) population-level negative impact. For each of our three primary outcomes, we examined the extent to which the substances (use disorder prevalence, individual-level negative impact, and population-level negative impact) significantly differed from each other. Means and standard errors were produced for each SUD by region, by respondent type, and for the overall sample. In the Results tables, overall means not sharing any superscript letter are significantly different by the *t*-test at the 5% level of significance [44]. Mean differences between regions and respondent type were tested by the *t*-test using Bonferroni-corrected alpha levels of 0.008 and 0.017 for region and respondent type, respectively. We reported *p*-values and Cohen’s *d* values.

To assess the quality of our results, we examined rates of missing data and conducted several reviews of the data. Rates of missing data were low (3.7% for alcohol, 3.3% for cannabis, 3.1% for cocaine, 6.1% for methamphetamines, and 4.4% for opioid). To verify the results were representative, we conducted three reviews. First, to ensure the results were not driven by one ASO, region, or respondent type, we assessed the distribution of respondents by each characteristic. Our review of respondents by ASO found the mean number of respondents per ASO was 2.3. This mean did not appreciably change when looking by region or respondent type (Table 1). This indicates that no one ASO had an outsized influence on the estimates. Second, we reviewed the distribution of responding ASOs by region, and found it was in line with the distribution of all ASOs invited to participate in the study. Third, we reviewed the distribution of respondents by region and respondent type. We determined the respondents were reasonably distributed based on each region and respondent type.

**Table 1:** Number of participating organizations and respondents by region and perspective

	All	Region								Stakeholder Perspective					
		Northeast		South		Midwest		West		Clients with HIV		ASO Staff		HPC members	
	N	n	(%)	n	(%)	n	(%)	n	(%)	n	(%)	n	(%)	n	(%)
Number of organizations	282	66	(23.4)	98	(34.8)	43	(15.2)	75	(26.6)	65	(23.0)	215	(76.2)	59	(20.9)
Total number of respondents	643	127	(19.8)	227	(35.3)	123	(19.1)	166	(25.8)	109	(17.0)	419	(65.2)	115	(17.9)

ASO = HIV/AIDS Service Organization; HPC = HIV Planning Council; Many ASOs had multiple clients and staff participate, which is why the number of organizations for respondent type are greater than the number of unique organizations

## Results

### Participants

**Fig. 1:**
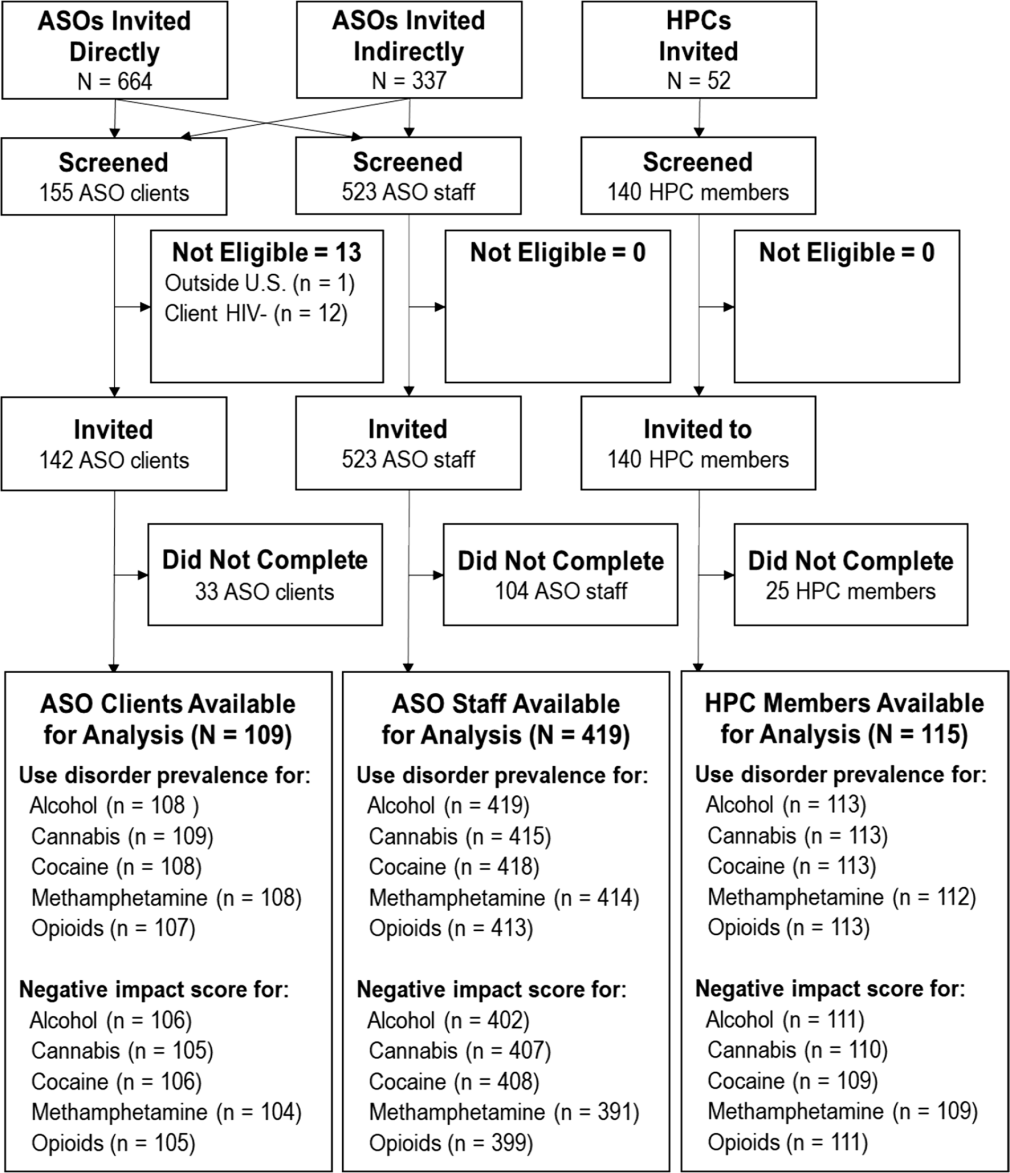
Participant Flowchart

As shown in Fig. 1, we directly invited 664 ASOs and 52 HPCs to have staff and clients complete the project’s informed consent form and screener; 337 ASOs were invited indirectly through other means (e.g. being forwarded the invitation from an invited ASO). Overall, 819 clients with HIV, ASO staff, and HPC members completed the screener and 805 were eligible to participate. Of the 805 total participants invited, 643 (80%) participated in the Real-Time Delphi. Based on the screener data, there were no significant (*p* < 0.05) differences between the 643 participants and the 162 who did not participate.

As shown in Table 1, 643 participants representing 282 unique ASOs/HPCs were included in these analyses. Most participants (65.2%, n = 419) were ASO staff. The South was the most represented region both in terms of number of ASOs/HPCs (34.8%, n = 98) and number of participants (35.3%, n = 227). Among all participants, the mean age was 43.6 years (*SD* = 12.4). The majority of participants identified as female (52.1%, n = 335); 41.7% (n = 268) were male, 5.1% (n = 33) were transgender, genderqueer, or gender-non-conforming. The majority of participants were white (58.9%, n = 379). Approximately one-third of participants identified as black or African American (36.4%, n = 234) and approximately one quarter (23.2%, n = 149) identified as Hispanic or Latino. In terms of lifetime SUDs (i.e. ever met two or more of the 11 DSM-5 criteria during a 12-month period), 46.8% (n = 301) reported lifetime alcohol use disorder, 30.8% (n = 198) reported lifetime cannabis use disorder, 21.3% (n = 137) reported lifetime cocaine use disorder, 13.7% (n = 88) reported lifetime methamphetamine use disorder, and 11.4% (n = 73) reported lifetime opioid use disorder.

### Prevalence

Table 2 Panel A includes the mean and standard error regarding perceived SUD prevalence among PWH, reported by (i) the four U.S. regions (Northeast, South, Midwest, and West), (ii) the three stakeholder perspectives (clients with HIV, ASO staff, and HPC members), and (iii) overall. Cannabis and alcohol use disorders were perceived as the most prevalent SUDs overall (42.3% and 41.9%, respectively), followed by opioid use disorder (34.6%), methamphetamine use disorder (32.2%), and cocaine use disorder (28.1%), most of which were significantly different from one another in terms of magnitude. Table 2 Panel B summarizes the regional and stakeholder perspective differences identified in perceived SUD prevalence and includes the *p* value and the effect size (Cohen’s *d*; 0.2 = small effect, 0.5 = medium effect, 0.8 = large effect) [45]. The largest difference (*d* = 0.73) was between the perceived prevalence of methamphetamine use disorders among PWH in the West (40.8%) and the Northeast (24.2%). Regarding the perceived prevalence of alcohol use disorders, the only significant difference found was the small difference (*d* = 0.32) between the South (44.1%) and the West (37.1%). In terms of the prevalence of cannabis use disorders, there were medium-sized differences between the West (35.7%) and both the Northeast (47.0%; *d* = 0.42) and the South (44.4%; *d* = 0.33). For cocaine use disorders, the average perceived prevalence for the West (19.5%), was significantly lower (medium-sized effects) compared to each of the other three regions. Additionally, ASO staff reported significantly lower cocaine use disorder prevalence (25.1%) than both HPC members (31.6%; *d* = 0.30) and clients with HIV (36%; *d* = 0.49). For methamphetamine use disorders, the West’s 40.8% average rate was also significantly higher than the average in the South (28.6%; *d* = 0.55), and ASO staff again reported a significantly lower prevalence of 29.5% compared to clients with HIV (38.9%; *d* = 0.40). Last, the Northeast’s prevalence for opioid use disorders (41.3%) had small-to-medium effect size differences with both the South (32.0%; *d* = 0.40) and the West (35.0%; *d* = 0.35).

**Table 2:** Prevalence of substance use disorder by region and stakeholder perspective

Panel A. Descriptive Statistics																
Use Disorder	Region								Stakeholder Perspective						Overall	
	Northeast		South		Midwest		West		Clients with HIV		ASO Staff		HPC Member			
	Mean	(SE)	Mean	(SE)	Mean	(SE)	Mean	(SE)	Mean	(SE)	Mean	(SE)	Mean	(SE)	Mean	(SE)
Alcohol	43.0	(2.03)	44.1	(1.54)	43.2	(1.96)	37.1	(1.56)	42.0	(2.43)	41.8	(1.07)	42.2	(1.92)	41.9^a^	(0.88)
Cannabis	47.0	(2.34)	44.4	(1.74)	42.4	(2.45)	35.7	(2.09)	46.0	(2.70)	40.1	(1.29)	46.6	(2.50)	42.3^a^	(1.06)
Cocaine	31.3	(2.18)	31.1	(1.50)	30.8	(2.26)	19.5	(1.41)	36.0	(2.59)	25.1	(1.02)	31.6	(2.26)	28.1	(0.91)
Methamphetamine	24.2	(2.09)	28.6	(1.45)	35.3	(2.31)	40.8	(1.77)	38.9	(2.52)	29.5	(1.11)	35.5	(2.28)	32.2	(0.95)
Opioid	41.3	(2.47)	32.0	(1.38)	35.4	(2.07)	32.5	(1.82)	36.4	(2.42)	33.3	(1.12)	37.5	(2.27)	34.6	(0.93)

Panel B. Significant Differences and Effect Sizes										
Use Disorder	Comparisons by Region					Comparisons by Stakeholder Perspective				
	Groups			p-value	Cohen’s *d*	Groups			p-value	Cohen’s *d*
Alcohol	South	>	West	0.002	0.32	*No significant differences by perspective*				
Cannabis	Northeast	>	West	< 0.001	0.42	*No significant differences by perspective*				
	South	>	West	0.002	0.33					
Cocaine	Northeast	>	West	< 0.001	0.56	Clients with HIV	>	ASO Staff	< 0.001	0.49
	South	>	West	< 0.001	0.56	HPC Members	>	ASO Staff	0.009	0.30
	Midwest	>	West	< 0.001	0.53					
Methamphetamine	Midwest	>	Northeast	< 0.001	0.46	Clients with HIV	>	ASO Staff	< 0.001	0.40
	West	>	Northeast	< 0.001	0.73					
	West	>	South	< 0.001	0.55					
Opioid	Northeast	>	South	0.001	0.40	*No significant differences by perspective*				
	Northeast	>	West	0.004	0.35					

ASO = HIV/AIDS Service Organization; HPC = HIV Planning Council. Impact scores are the sum of eight items on a 4-point scale, with an index rate from 0 (selecting “0 = no negative impact at all”) to 24 (selecting “3 = a major negative impact” for all items). In Panel A, overall means not sharing any superscript letter are significantly different by the *t*-test at the 5% level of significance. In Panel B, mean differences between regions and respondent type were tested by the *t*-test using Bonferroni-corrected alpha levels of .008 and .017 for region and respondent type, respectively

### Individual-Level Negative Impact Scores

**Fig. 2:**
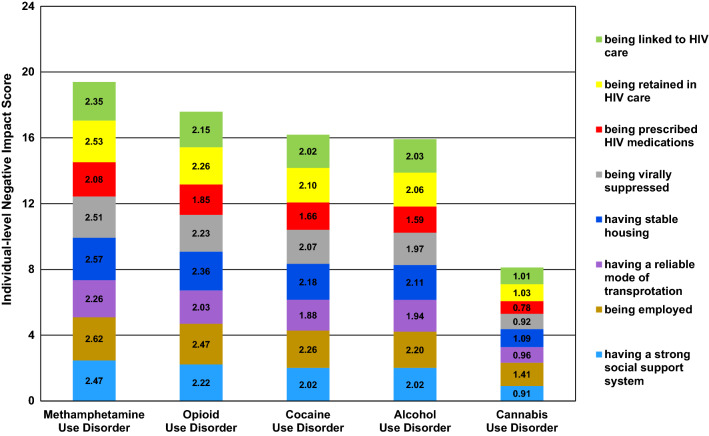
Contribution of each indicator to the individual-level negative impact scores

Figure 2 visualizes the average individual-level negative impact score for each of the five SUDs, as well as how each of the HIV care continuum indicators (being linked to HIV care, being retained in HIV care, being prescribed HIV medications, being virally suppressed) and other important areas of life (having stable housing, having a reliable mode of transportation, being employed, and having strong social support system) contributed to each SUD’s individual-level negative impact score. Negative impact scores on the HIV care continuum for methamphetamine use disorder fell between “moderate” and “major” for being linked to HIV care, being retained in HIV care, and being virally suppressed; means were similar for having stable housing, being employed, and having a strong social support system. For opioid, cocaine, and alcohol use disorders, the negative impact scores on being linked to HIV care, being retained in HIV care, and being virally suppressed had means near 2.0, representing “moderate” negative impacts. Means for being prescribed HIV medications tended to be lower, with means less than 2.0. In contrast to the other SUDs, respondents rated the impacts of cannabis use disorder on the four elements of the HIV care continuum and the four other important areas of life, on average, as generally “minor” with means from 0.78 to 1.41.

For each SUD, the average individual-level negative impact score is provided as part of Table 3 Panel A, with this information again reported by region, stakeholder perspective, and overall. Overall, methamphetamine use disorder was perceived as having the greatest individual-level negative impact score (19.4), followed by opioid use disorder (17.6), cocaine and alcohol use disorders (16.2 and 15.9, respectively), and cannabis use disorder (8.1). Based on the adjusted analyses, which again controlled for region and perspective, the individual-level negative impact scores between alcohol (15.9) and cocaine use disorders (16.2) did not differ significantly. In contrast, all other pairwise comparisons between individual-level negative impact scores were significantly different. The absolute effect size difference was very large between cannabis use disorder and use disorders for methamphetamine (*d* = 2.17), opioids (*d* = 1.77), alcohol (*d* = 1.63), and cocaine (*d* = 1.51). On average, the remaining significant differences were close to medium sized (average *d* = 0.45).

**Table 3:** Individual-level negative impact score by region and stakeholder perspective

Panel A. Descriptive Statistics																
Use Disorder	Region								Stakeholder Perspective						Overall	
	Northeast		South		Midwest		West		Clients with HIV		ASO Staff		HPC Member			
	Mean	(SE)	Mean	(SE)	Mean	(SE)	Mean	(SE)	Mean	(SE)	Mean	(SE)	Mean	(SE)	Mean	(SE)
Alcohol	16.4	(0.39)	16.0	(0.28)	15.7	(0.36)	15.6	(0.36)	16.1	(0.20)	15.5	(0.46)	15.4	(0.44)	15.9^a^	(0.17)
Cannabis	8.3	(0.47)	8.9	(0.35)	8.0	(0.40)	7.1	(0.38)	7.9	(0.24)	8.5	(0.55)	8.5	(0.48)	8.1	(0.20)
Cocaine	17.1	(0.44)	17.1	(0.33)	16.3	(0.50)	14.2	(0.49)	16.0	(0.27)	17.0	(0.51)	16.2	(0.54)	16.2^a^	(0.22)
Methamphetamine	18.4	(0.56)	19.4	(0.35)	20.1	(0.40)	19.7	(0.41)	19.3	(0.25)	19.7	(0.58)	19.4	(0.47)	19.4	(0.21)
Opioid	18.4	(0.51)	17.7	(0.35)	17.8	(0.50)	16.6	(0.46)	17.6	(0.27)	17.6	(0.58)	17.4	(0.54)	17.6	(0.22)

Panel B. Significant Differences and Effect Sizes								
Use Disorder	Comparisons by Region					Comparisons by Stakeholder Perspective		
	Groups			p-value	Cohen’s *d*	Groups	p-value	Cohen’s *d*
Alcohol	*No significant differences by region*					*No significant differences by perspective*		
Cannabis	South	>	West	< 0.001	0.36	*No significant differences by perspective*		
Cocaine	Northeast	>	West	< 0.001	0.51	*No significant differences by perspective*		
	South	>	West	< 0.001	0.52			
	Midwest	>	West	0.003	0.35			
Methamphetamine	*No significant differences by region*					*No significant differences by perspective*		
Opioid	Northeast	>	West	0.008	0.32	*No significant differences by perspective*		

ASO = HIV/AIDS Service Organization; HPC = HIV Planning Council. Population-level means were calculated by multiplying the individual-level impact by perceived prevalence rate. In Panel A, overall means not sharing any superscript letter are significantly different by the *t*-test at the 5% level of significance. In Panel B, mean differences between regions and respondent type were tested by the t-test using Bonferroni-corrected alpha levels of .008 and .017 for region and respondent type, respectively

For both alcohol use disorder and methamphetamine use disorder, there were no significant differences between regions or stakeholder perspectives (see Table 3 Panel B). Furthermore, there were no significant differences between stakeholder perspectives for any of the SUDs. There were significant regional differences for the other three SUDs. Respondents from the South perceived cannabis use disorder as having a significantly more negative impact (17.1) than the West (7.1, *d* = 0.36). Respondents in the Northeast perceived opioid use disorder as having a significantly more negative impact (18.4) than in the West (16.6, *d* = 0.32). Finally, respondents in the West perceived cocaine use disorder as having a significantly more negative impact (14.2) than in the Midwest (16.3; *d* = 0.35), Northeast (17.1; *d* = 0.51), and South (17.1; *d* = 52).

### Population-Level Negative Impact Scores

Table 4 Panel A includes the means and standard errors for each SUD’s population-level negative impact score by region, perspective, and overall. Again, population-level negative impact scores were computed by weighting (i.e. multiplying) each respective SUD’s individual-level negative impact score by its perceived prevalence. Alcohol use disorder, methamphetamine use disorder, and opioid use disorder had the highest overall population-level negative impact scores (6.9, 6.5, and 6.4, respectively). These scores were not significantly different from one another. Scores for cocaine use disorder (5.0) and cannabis use disorder (3.7) were lower. All other pairwise comparisons were statistically different, with the difference between use disorders for alcohol and cannabis having the largest effect size (*d* = 0.72). Medium effect size differences were found between cannabis use disorder and use disorders for opioids (*d* = 0.64) and methamphetamines (*d* = 0.59*).* The remaining significant differences were small (average *d* = 0.31).

**Table 4:** Population-level negative impact score by region and stakeholder perspective

Panel A. Descriptive Statistics																
Use Disorder	Region								Stakeholder Perspective						Overall	
	Northeast		South		Midwest		West		Clients with HIV		ASO Staff		HPC Member			
	Mean	(SE)	Mean	(SE)	Mean	(SE)	Mean	(SE)	Mean	(SE)	Mean	(SE)	Mean	(SE)	Mean	(SE)
Alcohol	7.2	(0.41)	7.4	(0.32)	6.9	(0.36)	6.1	(0.32)	6.9	(0.22)	7.0	(0.48)	6.8	(0.39)	6.9^a^	(0.18)
Cannabis	4.2	(0.36)	4.2	(0.27)	3.6	(0.31)	2.8	(0.24)	3.5	(0.17)	4.2	(0.44)	4.2	(0.34)	3.7	(0.15)
Cocaine	5.6	(0.45)	5.7	(0.33)	5.3	(0.47)	3.2	(0.28)	4.4	(0.21)	6.6	(0.56)	5.4	(0.47)	5.0	(0.19)
Methamphetamine	4.6	(0.44)	5.9	(0.33)	7.2	(0.50)	8.3	(0.41)	6.0	(0.25)	8.1	(0.58)	7.0	(0.49)	6.5^a^	(0.21)
Opioid	7.8	(0.55)	5.9	(0.30)	6.6	(0.43)	5.8	(0.38)	6.2	(0.24)	6.7	(0.52)	6.8	(0.48)	6.4^a^	(0.20)

Panel B. Significant Differences and Effect Sizes										
Use Disorder	Comparisons by Region					Comparisons by Stakeholder Perspective				
	Groups			p-value	Cohen’s *d*	Groups			p-value	Cohen’s *d*
Alcohol	South	>	West	0.006	0.28	*No significant differences by perspective*				
Cannabis	Northeast	>	West	< 0.001	0.41	*No significant differences by perspective*				
	South	>	West	< 0.001	0.38					
Cocaine	Northeast	>	West	< 0.001	0.56	ASO Staff	>	Clients with HIV	< 0.001	0.48
	South	>	West	< 0.001	0.56					
	Midwest	>	West	< 0.001	0.49					
Methamphetamine	Midwest	>	Northeast	< 0.001	0.51	ASO Staff	>	Clients with HIV	< 0.001	0.41
	West	>	Northeast	< 0.001	0.74					
	West	>	South	< 0.001	0.48					
Opioid	Northeast	>	South	0.003	0.38	*No significant differences by perspective*				
	Northeast	>	West	0.003	0.38					

ASO = HIV/AIDS Service Organization; HPC = HIV Planning Council. In Panel A, overall means not sharing any superscript letter are significantly different by the *t*-test at the 5% level of significance. In Panel B, mean differences between regions and respondent type were tested by the t-test using Bonferroni-corrected alpha levels of .008 and .017 for region and respondent type, respectively

Significant differences by region or perspective are summarized in Table 4 Panel B. Again, the largest difference was between the population-level negative impact score for methamphetamine use disorders reported by respondents from the West (8.3) and Northeast (4.6; *d* = 0.74). For alcohol use disorders, the only significant difference was a small difference (*d* = 0.28) between respondents in the South (7.4) and West (6.1). For cannabis use disorders, scores for respondents in the Northeast and South (both 4.2) were significantly higher than the West (2.8). The population-level negative impact score for cocaine use disorder was significantly lower for respondents from the West (3.2) than from the Northeast (5.6), South (5.7) and Midwest (5.3). Additionally, the score for cocaine use disorder was significantly higher for ASO staff (6.6) than clients with HIV (4.4). For methamphetamine use disorders, the score from the West (8.3) was significantly higher than both the Northeast (4.6) and the South (5.9). Furthermore, ASO staff again reported a significantly higher score (8.1) than clients with HIV (6.0). Finally, for opioid use disorders, the score from respondents in the Northeast (7.8) was higher than the South (5.9) and West (5.8).

## Discussion

Building on the extant literature [5, 25, 28–30, 34, 38, 46, 47], we sought to advance knowledge regarding the prevalence, individual-level negative impacts, and population-level negative impacts of different SUDs among PWH in the U.S. The highest perceived rates of SUDs were for alcohol and cannabis use disorders. Similar to other U.S.-based research [5, 46], we identified several regional differences, with each of the five SUDs having at least one significant regional difference. These findings have important implications for efforts to develop national- and/or state-level policies. Generally, the perceived prevalence of alcohol, cannabis, cocaine, and opioid use disorders was lower in the West than in other regions, while the perceived prevalence of methamphetamine use disorder was greater in the West. These findings are somewhat different than U.S. population data where alcohol and illicit drug use disorder rates are relatively lower in the South and higher in the West [46]. Hartzler et al. also found wide variability in prevalence among PWH by site, but due to confidentiality issues did not identify the location of each site [5]. Additionally, although there was generally good agreement between the three unique perspectives examined, we found that the rates ASO staff provided for cocaine use disorder and methamphetamine use disorder were significantly lower than the rates provided by clients with HIV.

Just as cannabis use disorder was the most prevalent use disorder reported by Hartzler et al. (31%) [5], we found cannabis use disorder to be perceived as the most prevalent, albeit higher, at 42%. Among the possible explanations for this 11 percentage-point difference, one of the most likely is that marijuana-related policies and legislation have significantly changed (i.e. increased legalization for medical and recreational purposes) since the 2007–2014 time period when data for the other study was collected, and there are parallel significant increases in cannabis use disorder [48]. However, cannabis use disorder had the lowest population-level negative impact score across regions and stakeholders, which follows national population-based work showing declines in perception of risk of marijuana use (not SUD) in the general public [48]. This is a complex and unfolding issue. Cannabis use disorder has clear negative impacts on mental health and other areas [49], but may or may not have some medical benefits, including in HIV care [13]. Increasing quality of research on effects of medical marijuana and impacts of cannabis use disorders will hopefully shed more light on this issue.

Like Hartzler et al., we also found alcohol use disorder to be the second most prevalent. However, the 41.9% perceived prevalence we found was 2.2 times greater than their 19%. Notably, our estimate is essentially identical to the 42% prevalence that Duko et al. [25] found in their systematic review and meta-analysis of PWH in developed countries. An acknowledged limitation of the data examined by Hartzler et al., which may help explain these differences, is that patients appearing intoxicated were not administered the use disorder assessment. We believe that the remarkable level of agreement between the alcohol use disorder estimate provided by our study’s sample of stakeholders and Duko et al.’s meta-analysis supports the wisdom of crowds research showing that a crowd’s average response is quite accurate [50, 51]. Thus, there is good reason to have confidence in the accuracy of the prevalence estimates we found for the other four use disorders: 42% for cannabis use disorder, 35% for opioid use disorder, 32% for methamphetamine use disorder, and 28% for cocaine use disorder.

Although not specifically for PWH, nor within the U.S., nor for SUD, the negative impacts of different substances have been examined in the United Kingdom [29], European Union [30], and Australia [28], with each study identifying alcohol as the substance of greatest harm. For Australia, alcohol was followed by methamphetamine and opioids, with a substantial drop in the harm score for the other substances examined [28]. The other two studies each found the second and third most harmful substances, after alcohol, to be heroin and crack cocaine, with methamphetamine, cocaine, and tobacco grouped together much lower [29, 30]. As part of our study, we found methamphetamine use disorder to have the highest individual-level negative impact and opioid use disorder to have the second highest, with a significant difference between these two use disorders and each of the others. The individual-level negative impacts of use disorders for cocaine and alcohol were about the same, and both were significantly higher than cannabis use disorder. Several important differences make it challenging to directly compare our results to these other three studies, including geographic differences, the difference between substance use and a SUD, the difference between the general population and PWH, and the difference in the rating criteria (i.e. our criteria included indicators of the HIV care continuum). However, even with these key differences, there appears to be relatively good convergence that the perceived harms associated with use/use disorders for alcohol, opioids, and methamphetamines are among the highest of all substances. As research improves our understanding of the ways in which outcomes for PWH are impacted by substance use and SUDs, there is a need for greater specificity regarding the substance and/or SUD (i.e. the impairment caused by some substances are significantly greater than others). Future research could further expand the knowledge base by exploring the extent to which the negative impacts on the HIV continuum of care vary by severity of substance use, including severity of substance use disorder (i.e. if there is a dose response relationship).

Even separately, a SUD’s high prevalence among the population of PWH or its great negative impact on an individual with comorbid HIV and SUD can provide strong justification for efforts to provide integrated care for both conditions. However, the justification(s) for integrated care may be the strongest when both the individual-level negative impact of the SUD is weighted (i.e. multiplied) by the SUD’s prevalence to yield a population-level negative impact. Indeed, it is for this reason that Bonomo et al. [28] conducted supplementary analyses in which their harm scores were weighted using the substance’s prevalence in Australia. Although alcohol remained the most harmful substance, analyses using weighting indicated that cannabis had a substantially higher perceived harm score that was essentially the same as the weighted harm score for opioids.

Underlining the importance of using prevalence and negative impact estimates in combination, a key implication of our study is that conclusions drawn from the population-level negative impact scores may not always align with the conclusions that one may have drawn based on only one estimate. For example, based on prevalence alone, finite resources may be focused on addressing alcohol and cannabis use disorders, whereas only considering the individual-level negative impact scores would have suggested targeting resources toward methamphetamine and opioid use disorders. However, based on population-level negative impact estimates, we suggest that it may be best to focus finite resources on the *big three SUDs* among PWH in the U.S.—alcohol use disorder, methamphetamine use disorder, and opioid use disorder. Focusing on these three substances is important, given that efforts to address the opioid crisis in the U.S. [52] currently overshadow efforts to address the country’s alcohol and methamphetamine crises.

It is time to not just call for integration of SUD services into HIV care but to take action. There are myriad barriers to HSOs integrating SUD care [53], and HIV care providers may find the task too large and overwhelming if the expectation is to integrate care for all SUDs. Focusing on specific SUDs that are most prevalent in specific patient populations, and thence to the specific treatments that are most effective for those SUDs (e.g. buprenorphine for opioid use disorder; a combination of psychosocial treatments for methamphetamine use disorder) may be more manageable for ASOs.

Funders and policymakers need to be aware of results such as ours as well, so that they can design funding streams and policies that match regional or local community needs. The *Ending the HIV Epidemic* plan [2] brings resources to jurisdictions where HIV transmission is highest. Addressing specific SUDs that are most prevalent and that have the highest negative impact on PWH within those jurisdictions is necessary to the plan’s success. This is true at the national level through agencies including the Centers for Disease Control and Prevention (CDC) and Health Resources and Services Administration (HRSA), but also at the regional and local level through HPCs.

In addition to having several important strengths (e.g. large sample size, diverse sample in terms of both geographical region of the U.S. and perspective, high response rate), our study has several important limitations. First, our study examined the prevalence and negative impacts of different SUDs for PWH in the U.S. As such, it is currently unknown to what extent our findings may generalize to PWH from other countries or to the general population within the U.S. Second, our study’s prevalence estimates were based on stakeholders’ perceptions of PWH in their area. However, we believe that wisdom of crowds research [50, 51] increases confidence in this method, which seems to be further supported given the highly similar estimate for SUD between our sample and Duko et al. [25]. Third, our study did not assess the extent to which each SUD may cause harm to others (e.g. injuries to passengers during substance-involved motor vehicle accidents). We believe this limitation does complicate comparisons with some prior research investigating the impact of different substances [28–30]. Fourth, our study’s individual-level negative impact estimates did not include the SUD’s negative impact on HIV medication adherence. Although we had considered including HIV medication adherence, we ultimately decided against its inclusion given that it is not one of the HIV continuum of care indicators, and it is highly correlated with viral suppression, an HIV continuum of care indicator that was included in the study. Fifth, our study did not include tobacco or amyl nitrite (poppers). While both were considered for inclusion as part of this study, they were excluded as an effort to minimize participant burden and improve comparability of our findings with Hartzler et al. [5].

## Conclusions

Similar to the study conducted by Hartzler and colleagues, this study advances knowledge regarding the prevalence of the “American SUD-HIV syndemic” [5]. Moreover, this study advances knowledge regarding the individual-level and population-level negative impacts of five different SUDs on PWH within the U.S. In addition to further demonstrating the need for integrating SUD services within HIV settings [1, 5, 54–58], our findings may help national-, state-, and county-level policymakers, HPCs, and ASOs refine how their finite funding is allocated for addressing the HIV-SUD syndemic within the U.S.
